# From PFOS to a Sum of 25 PFASs: Regulatory and Effect-Based Risk Assessment in the River Ruhr

**DOI:** 10.3390/toxics14100866

**Published:** 2026-09-29

**Authors:** Gerhard Schertzinger, Raquel Gonzalez de Vega, Viktoria Müller, Thomas Riedel, Florian Zaun, Jörg Feldmann

**Affiliations:** 1 IWW Institut für Wasserforschung Gemeinnützige GmbH, Moritzstr. 26, 45476 Mülheim an der Ruhr, Germany; 2 TESLA—Analytical Chemistry, Institute of Chemistry, University of Graz, Universitätsplatz 3, 8010 Graz, Austria; 3 James Hutton Institute, Craigiebuckler, Aberdeen AB15 8QH, Scotland, UK

**Keywords:** PFAS, water framework directive, environmental quality standard, PFAS CALUX, effect-based monitoring, extractable organic fluorine

## Abstract

Regulatory assessment of per- and polyfluoroalkyl substances (PFASs) in European surface waters is shifting from a single-compound environmental quality standard (EQS) for perfluorooctane sulfonic acid (PFOS) toward a potency-weighted sum of 25 PFASs criterion under the revised Water Framework Directive (WFD). This environmental survey investigated how this regulatory change affects screening-level assessment of PFAS contamination and whether chemical classifications are consistent with an effect-based endpoint. Fourteen surface-water samples from the river Ruhr and its tributary, the Möhne (Germany), were enriched by weak anion-exchange solid-phase extraction and analyzed by targeted liquid chromatography-electrospray ionization-mass spectrometry, combustion ion chromatography for extractable organic fluorine (EOF), and the TTR–TRβ chemical activated luciferase gene expression bioassay (referred to as PFAS CALUX). Thirteen PFAS were quantified. At every location with valid target-PFAS data except site A, both the former PFOS annual average EQS of 0.65 ng L^−1^ and the revised water-column criterion of 4.4 ng PFOA-equivalents L^−1^ for the sum of 25 PFASs were exceeded when the latter was calculated from the WFD-listed PFASs covered by the analytical method. Because trifluoroacetic acid and perfluor (((5-methoxy-1,3-dioxolan-4-yl)oxy)acetic acid) were not measured, this represents a lower-bound estimate of the revised metric. In contrast, the PFAS CALUX bioassay exceeded the pragmatic effect-based trigger value in 10 of 14 samples and produced discordant classifications at several sites. Target PFASs explained <5% of EOF and generally <3% of the observed bioassay response. Thus, in this river dataset, expansion from PFOS alone to a potency-weighted PFAS mixture did not materially change the chemical screening classification, whereas effect-based assessment revealed a different spatial pattern. EOF provides complementary evidence of a substantial uncharacterized organofluorine burden. However, compared to other studies, it did not provide any insights into PFAS hotspots.

## 1. Introduction

Per- and polyfluoroalkyl substances (PFASs) constitute a large and chemically diverse class of synthetic organofluorine compounds, with more than 10,000 individual structures currently assumed to be in use or present as environmental transformation products. Their exceptional thermal and chemical stability has made PFASs valuable for numerous industrial applications and consumer products, including surface treatments, firefighting foams, textiles, food contact materials, and fluoropolymer production. However, these same characteristics also render PFASs extremely persistent, mobile, and prone to long-range environmental transport. With increasing detection in water, soil, air, wildlife, and humans, PFAS contamination has become a global concern. Beyond persistence, key properties such as bioaccumulation, toxicity, and the formation of transformation products raise further regulatory and public health challenges [1,2]. One of the greatest challenges lies in the wide variation in chemical structures within the PFASs group; for example, between short-chain and long-chain compounds. This results in significant differences in properties and environmental behavior [3], as well as in the specific requirements for appropriate analytical methods for environmental monitoring and risk assessment among the group of PFASs.

Monitoring and regulatory assessment of PFASs in aquatic systems have traditionally relied on targeted chemical analysis of a limited set of well-characterized compounds. Under the previous European Union (EU) Water Framework Directive (WFD), perfluorooctane sulfonic acid (PFOS) was assessed using an annual average environmental quality standard (AA-EQS) of 0.65 ng L^−1^ for inland surface waters [4,5]. The 2022 proposal to revise European water legislation [6] culminated in Directive (EU) 2026/805, which introduced an environmental quality standard (EQS) for ‘PFASs—sum of 25′ in surface waters, expressed as perfluorooctanoic acid (PFOA) equivalents using compound-specific relative potency factors (RPFs) [7]. The AA-EQS is 4.4 ng PFOA-equivalents L^−1^ (PFOA-eq.). This regulatory transition raises a practical environmental-monitoring question: does moving from a single-substance EQS for PFOS to a broader potency-weighted PFAS mixture criterion materially change the identification of sites of concern?

It is already well established that targeted PFAS analysis can account for only a fraction of the extractable organofluorine (EOF) present in environmental samples [8,9,10,11,12]. Reported comparisons between EOF and quantified PFASs frequently leave a large unidentified fluorine fraction, and effect-based studies likewise show biological responses that cannot be fully attributed to monitored PFASs [13]. Accordingly, the analytical blind spot itself is not treated as the principal novelty of the present study. Instead, here EOF is used first, as additional line of evidence to better understand spatial distribution of PFASs, and second, to examine whether it can be used to identify PFAS hotspots along the Ruhr River, as other studies have already shown [9].

Effect-based methods provide an orthogonal line of evidence because they integrate biological activity across complex mixtures rather than relying on a predefined chemical target list. Disruption of thyroid-hormone transport is a documented mode of action for several PFASs, including competitive binding to the thyroxine hormone (T4) transport protein transthyretin (TTR) [13,14,15]. Corresponding methods [13] capture not only known PFASs but also unknown substances and those present in low concentration, including mixture interactions. This may result in significant effects even if concentrations of single PFASs are below their respective effect threshold (“something from nothing effect”) [16]. Comparison with chemically derived PFOA equivalents therefore provides a means of evaluating whether a regulatory target-based classification is consistent with a mechanism-based biological response.

The river Ruhr is an important freshwater body in western Germany, supplying drinking water to more than five million people and receiving diffuse and point-source discharges from urban, industrial, and agricultural areas [17]. Target monitoring of selected PFAS compounds has been conducted over the past two decades as a precautionary measure and to track the evolution of concentrations following the illegal disposal of PFAS-containing material on farmland in the watershed since 2006 [17]. Thus, the river provides an insightful case study. In particular, it allows to examine how the stricter EU assessment framework for PFASs—which also includes compounds that have not been monitored before—changes the interpretation of previously recorded contamination patterns, and how this interpretation compares to an effect-based assessment.

In this study, we used an integrated analytical dataset from the river Ruhr and its tributary, the Möhne, primarily to evaluate the consequences of the transition from a PFOS-based assessment to the revised potency-weighted PFAS mixture approach. Target PFASs were quantified by liquid chromatography-electrospray ionization tandem mass spectrometry (LC-ESI-MS/MS), EOF was measured by combustion ion chromatography (CIC), and thyroid-hormone transport-disrupting activity was evaluated using the PFAS CALUX bioassay. The primary comparison was the site classification obtained using the former PFOS AA-EQS, the revised sum-of-25-PFASs water EQS calculated from the WFD-listed compounds covered by our method, and the PFAS CALUX effect-based trigger value (EBT).

The specific objectives were to

1. Characterize the occurrence and spatial distribution of target PFASs along the Ruhr river system as the basis for an environmental survey.
2. Determine whether screening-level site classification changes when moving from the former PFOS AA-EQS to the revised potency-weighted sum-of-25-PFASs water EQS.
3. Compare the chemical EQS-based classifications with the PFOA-BEQ response from PFAS CALUX and its pragmatic EBT.
4. Use EOF and chemically derived PFOA equivalents as complementary lines of evidence to quantify the analytical and effect gaps that remain outside the regulatory target-based assessment and to better understand spatial distribution of PFAS.
5. Evaluate whether EOF can be used for PFAS hotspot detection at the river Ruhr.

This framing distinguishes between two questions: the regulatory consequence of expanding the chemical target framework and the biological information provided by an effect-based method. It therefore treats the study primarily as an environmental survey and risk-screening comparison, while EOF is used to visualize the wider uncharacterized organofluorine burden as well as potential hotspots.

## 2. Materials and Methods

### 2.1. Study Site

The study site is in the federal state of North-Rhine-Westphalia, Germany. The source of the river Ruhr is located in a mountainous region, which is largely forested. The river flows over approx. 219 km westward before it eventually flows into the river Rhine. The first few kilometers of the catchment are characterized by forest and agriculturally used land. Further downstream the fraction of urban land use increases.

The river Ruhr receives several discharges along its flow path. In all, 304 direct dischargers, including 17 municipal wastewater treatment plants (WWTP), industrial discharges as well as rain induced discharges from combined and separate systems, are recorded in the official database of the Federal Ministry for the Environment [18]. The mean annual discharge (MQ) of the river Ruhr at the gauging station in Mülheim, close to the river Rhine, is approximately 73 m^3^ s^−1^ [19].

### 2.2. Sampling of Surface Water

**Figure 1:**
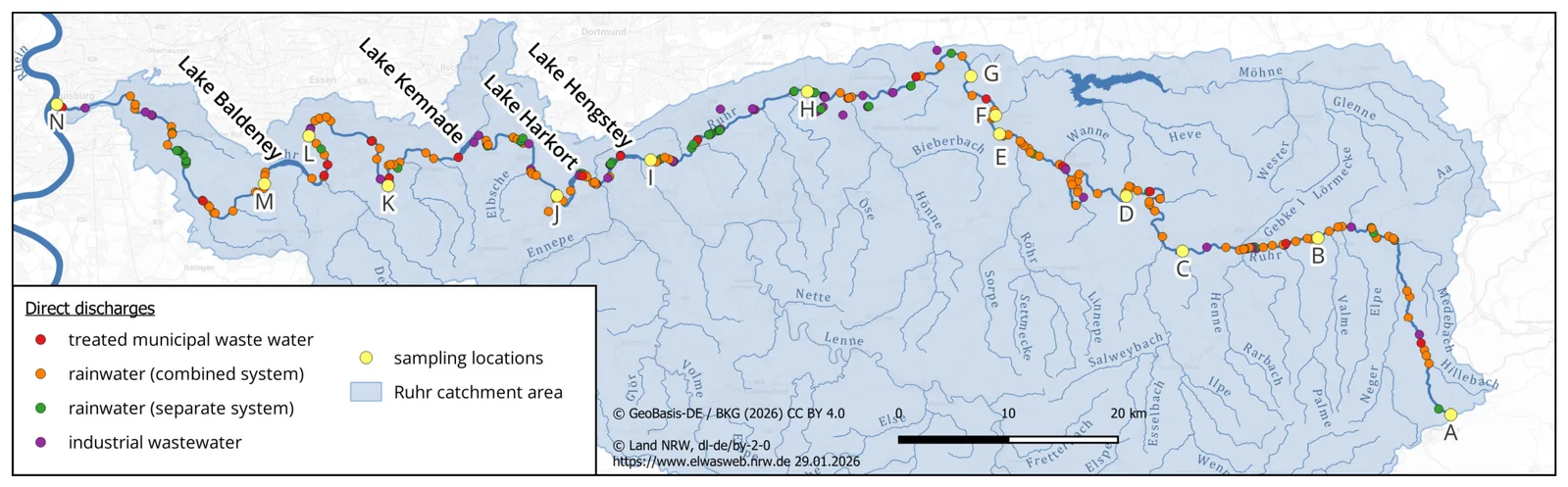
Catchment area of the river Ruhr includes sampling locations (A–N), as well as point sources such as municipal and industrial wastewater discharges or rain-induced discharges from separate and combined sewer systems.

Surface water samples from the river Ruhr in Western Germany were collected in two sampling campaigns. In total, fourteen samples were collected: thirteen samples along the flow section of the river Ruhr, from the source to the estuary (river Rhein at Duisburg harbor), as well as one sample from the tributary Möhne. Sampling locations correspond approximately to sampling locations used for regular quality monitoring on organic trace substances along the river Ruhr by the official authorities [17] and are described in detail in Table A1 (Appendix A), including sample-ID, sampling location, coordinates (latitude, longitude), surface water, river kilometer, date of sampling, and corresponding location of the regular Ruhr monitoring on organic trace substances. Samples were collected in May 2024 (Figure 1).

During sampling, water level (W), and discharge (Q) at the gauging station in Wetter (sampling location J, 79.75 km from estuary, right, Nr.: 2769133000200) were W = 289 cm and Q = 134 m^3^ s^−1^ on 22 May 2024 and W = 223 cm and Q = 64.8 m^3^ s^−1^ on 29 May 2024 [19].

At each sampling location two amber glass bottles of 2 L were rinsed two times with surface water (or deionized water). The bottles were previously washed in a laboratory dishwasher using dish soap for laboratory use and then rinsed with deionized water. Afterwards the bottles were filled completely with surface water or deionized water (field blank) and stored at 4 °C for no more than 10 days, until sample preparation via weak anion exchange solid phase extraction (WAX-SPE). Because this study included EOF and effect-based analyses, we prioritized minimizing the introduction of organofluorine-containing background from plastic materials [20]. We therefore selected glass containers despite the environmental protection agency’s recommendations for targeted PFAS methods. On-site, the parameters temperature, pH, and conductivity were measured in situ at each sampling location.

### 2.3. Sample Preparation by Weak Anion Exchange Solid Phase Extraction (WAX-SPE)

Surface water samples were prepared for analysis by weak anion exchange solid phase extraction (WAX-SPE). The procedure was adapted from [21], with slight modifications. The same basic procedure was used for EOF, target-PFAS analysis, and the PFAS CALUX bioassay. The individual SPE steps (conditioning, sample volume loaded, washing, elution, reconstitution) with method specific differences in loaded sample volume and final reconstitution are given in Table A2 (Appendix A). Clean up, extraction and preconcentration of the surface water samples were performed using Oasis^®^ WAX SPE cartridges (150 mg Sorbent, 6 cc, Waters GmbH, Eschborn, Germany, product no.: 186002493). Samples were loaded at approximately 2 drops s^−1^. Eluates were evaporated to dryness under nitrogen. The resulting concentration factors were 500 for EOF, 1000 for target PFASs, and 20,000 for PFAS CALUX. For chemical analysis, reconstituted extracts were centrifuged at 13,000 rpm for 5 min and the supernatants transferred to LC vials.

### 2.4. Targeted PFAS Analysis

Quantification of PFAS in surface water samples was performed by LC-ESI-MS/MS following WAX-SPE. The applied SPE-LC-MS/MS workflow has previously been established and successfully applied in our laboratory for PFAS analysis in environmental water samples [10,22]. In these earlier studies, the method demonstrated satisfactory recoveries, reproducibility, and analytical performance for the investigated PFAS compounds. Therefore, the analytical methodology used here is considered reliable and robust for the intended screening and comparative purposes of this study.

The target compounds and their relative potency factors are listed in Table A3 (Appendix A). PFASs were separated by reversed-phase liquid chromatography using an1260 Infinity II HPLC system coupled to an Ultivo LC/TQ (both Agilent Technologies Inc., Santa Clara, CA, USA) operated in negative multiple-reaction-monitoring mode. Details of the analytical column, gradient elution conditions, and monitored quantifier and qualifier transitions are provided in Table A4 and Table A5 (Appendix A).

For quality control, two 1 L bottles were filled with ultra-pure water. One of these was spiked with 10 μL of 1 mg L^−1^ PFAS-30-PAR standard mix and used for analyte recovery, while the other remained non-spiked and was used as laboratory method blank. Both ultra-pure water samples were then spiked with 10 µL of 16–40 µg L^−1^ MPFAC-HIF-ES mix and were subjected to the same sample preparation method (WAX-SPE) as the surface water samples. If observed, average area of the method blanks was subtracted from the samples. A calibration curve with seven points, ranging from 0.003 ng g^−1^ to 44.4 ng g^−1^ in methanol, was measured during the run. Quantification was performed using the analyte isotopically labelled internal standard ratio. Calibration curves were fitted using 1/x weighting. Analytical accuracy was evaluated using back-calculated standard concentrations and percentage accuracy at each calibration point. Calibration drift was additionally assessed through repeated measurements during the runtime using the 0.348 and 0.707 ng g^−1^ calibration points (number of replicates = 7). The coefficients of determination (R^2^), back-calculated accuracies and calibration-drift results are reported in Table A6 (Appendix A). The instrumental limit of quantification and detection (LOQ and LOD) were calculated as ten times and three times the signal to noise ratio of the linear regression divided by the slope, respectively. Method LOQ and LOD, reported in Table A3 (Appendix A), were calculated by multiplying the respective value with the dilution factors of 1000 derived from the sample preparation and dividing this by the average sample mass.

### 2.5. Extractable Organic Fluorine (EOF) Analysis

EOF was determined by CIC following WAX-SPE. A 100 µL aliquot of SPE eluate was injected and combusted using the procedure described previously [22,23]. Briefly, samples were introduced into a combustion furnace maintained at 1100 °C, where organofluorine compounds were quantitatively converted to hydrogen fluoride [23]. Analyses were performed using a Mitsubishi CIC system equipped with an ASC-270LS autosampler, an AQF-2100H combustion furnace, and a GA-211 gas absorption unit (all Nittoseiko Analytech Co., Ltd., Kyoto, Japan, formerly Mitsubishi Chemical Analytech). Argon and oxygen were supplied at flow rates of 200 and 400 mL min^−1^, respectively. The combustion unit was coupled to a Dionex Aquion RFIC ion chromatography system equipped with a Dionex IonPac AG19 guard column (2 × 50 mm) and a Dionex IonPac AG20 analytical column (2 × 250 mm) (all Thermo Fisher Scientific Inc., Waltham, MA, USA). Chromatographic separation was achieved using a potassium hydroxide gradient ranging from 3 to 30 mM. The column temperature was maintained at 30 °C, and the total run time was 25 min. Quantification was performed using a linear six-point sodium fluoride calibration from 0.1 to 10 µg mL^−1^ (r = 0.9996). The LOD and LOQ, calculated as three and ten times the standard deviation of the blank divided by the calibration slope, were 0.045 and 0.15 µg mL^−1^, respectively. Method reproducibility was evaluated by repeated injections of a 1 µg mL^−1^ sodium fluoride standard and yielded a relative standard deviation of 0.5% (n = 3). A WAX-SPE method blank was analyzed in parallel and its signal was subtracted from each sample result.

### 2.6. Analysis of Thyroid Receptor Blocking by PFASs (PFAS CALUX)

The potential of PFASs, present in surface water samples, to disrupt the T4 transport was analyzed using the TTR-TRβ CALUX bioassay (TRβ = nuclear T4-receptor, CALUX = chemical activated luciferase gene expression, BioDetection Systems b.v., Amsterdam, The Netherlands) following PFAS-selective WAX-SPE enrichment. This bioanalytical method is also known as PFAS CALUX [13,14,24,25].

The TTR-TRβ CALUX bioassay represents a combination of the TTR-binding competition assay with a thyroid-receptor reporter assay (TRβ CALUX bioassay) for functional readout. For the TTR-T4 binding competition assay, serial dilutions of the sample and blank extracts as well as the reference compound PFOA were incubated with 50 µL TTR (0.044 μM) and 100 µL T4 (0.052 μM) in Tris-buffer (pH 8.0) overnight at 4 °C. Afterwards a Bio-Gel P-6DG (Bio-Rad Laboratories GmbH, Feldkirchen, Germany, product no.: 150–0739) column was used to separate TTR-bound and free T4. Following centrifugation for 1 min at 120 g, 140 µL of the collected fractions were mixed with 500 µL of serum-free exposure medium. To determine the amount of TTR-bound T4, the TRβ CALUX was performed. Therefore, cells were seeded with assay medium in 96-well plates and pre-incubated for 24 h. Then, sample and blank eluates from the TTR-binding assay were exposed under standardized conditions (5% carbon dioxide; 37 °C; 100% humidity) for another 24 h. Thereupon, cells were lysed and the substrate luciferin was added to analyze luciferase activity using a luminometer (Platereader, Tecan, Männedorf, Switzerland).

Each sample dilution series was tested in triplicate. Dose-response analysis was performed as described by Behnisch et al. 2021 [14]. The PFOA-BEQ was calculated based on the PC_80_ [26] using the software GraphPad Prism 10.5.0 (GraphPad Software, Boston, MA, USA). PC_80_ represents the concentration at which the activity of the sample equals 80% of the maximum response of the reference compound PFOA. For evaluation, only the data that fulfil the quality criteria, as described in detail in [27], were used.

### 2.7. Conversion of PFAS Concentrations to PFOA-Equivalent Concentrations (PFOA-EQ)

To compare the chemical data with both the revised WFD metric and the effect-based results, quantified PFAS concentrations were converted to PFOA equivalents using relative potency factors (RPFs) referenced to PFOA. For regulatory screening, the in vivo RPFs associated with the WFD sum-of-25-PFASs approach were used [7]; for comparison with the PFAS CALUX response, in vitro RPFs were used where available [15].

For comparison with the revised WFD water EQS of 4.4 ng PFOA-eq. L^−1^, PFOA equivalents were calculated for the WFD-listed PFAS covered by the analytical method using the corresponding in vivo RPFs [7]. Of the 25 PFASs in the revised metric, 23 were included in the target method; trifluoroacetic acid (TFA) and 2,2-difluoro-2-([2,2,4,5-tetrafluoro-5-(trifluoromethoxy)-1,3-dioxolan-4-yl]oxy)acetic acid (C6O4) were not analyzed. Up to 13 of the WFD-listed target compounds were quantified in individual samples. Results below the LOQ were not included in this calculation; no measured concentrations occurred between the LOD and LOQ. The resulting value is referred to as PFOA-TEQ_WFD_. Because two compounds in the regulatory sum were not measured, PFOA-TEQ_WFD_ should be interpreted as a lower-bound estimate of the complete sum-of-25-PFASs metric.

For direct comparison with PFOA-BEQ from PFAS CALUX, chemically derived PFOA equivalents were also calculated using in vitro RPFs. In vitro RPFs were available for 23 targeted compounds. For compounds that were part of this calculation but were not quantified, the respective LOQ was used as a conservative upper-bound surrogate for the possible target-PFAS contributions. This chemically derived estimate is referred to as PFOA-CEQ_23_.

### 2.8. Screening-Level PFAS Risk Assessment

The chemical and bioanalytical data were compared with their respective assessment criteria to evaluate screening-level indications of concern in the sampled surface water. This comparison is not intended to constitute a formal WFD chemical-status assessment, because this study represents discrete water sampling and does not cover all matrices, temporal requirements, or all 25 PFASs in the revised metric.

First, measured PFOS concentrations were compared with the former PFOS AA-EQS of 0.65 ng L^−1^ for inland surface waters [4]. Second, PFOA-TEQ_WFD_ at each location was compared with the revised annual average water EQS of 4.4 ng PFOA-eq. L^−1^ for the sum of 25 PFASs [7]. Because TFA and C6O4 were not measured, this second comparison is conservative with respect to the complete regulatory sum. Third, PFOA-BEQ was compared with the pragmatic PFAS CALUX EBT of 0.71 µg PFOA-eq. L^−1^, which was derived from the fifth percentile of a previously measured surface-water dataset and is used as starting point for PFAS risk assessment in surface waters using the PFAS CALUX bioassay and should be understood as a screening threshold rather than a scientifically sound EBT nor an regulatory criterion [24].

For each approach, the ratio of the measured or equivalent concentration to the respective assessment criterion was calculated. Ratios > 1 were classified as exceedances for the purpose of this comparative screening analysis.

## 3. Results

### 3.1. On-Site Parameters

At each sampling location (A–N), the on-site parameters temperature, conductivity, and pH were measured in situ (Table A7, Appendix B). The water temperature increased along the flow section of the river Ruhr from 8.8 °C at the source (A) to 18 °C at the estuary (N). The conductivity also increased downstream from 82 µS cm^−1^ (A) to 382 µS cm^−1^ (N). The pH in the surface water samples of the river Ruhr was constant and ranged between 7.1 and 7.8. The sampling location at the Ruhr tributary, river Möhne, showed a slightly lower water temperature as well as slightly higher conductivity and pH compared to the sampling locations at the river Ruhr, located before and after the tributary Möhne.

### 3.2. Target PFASs

Target-PFAS concentrations in the surface-water samples were either below the LOD or above the LOQ (Table A8, Appendix B). Recoveries of quantified PFASs ranged from 67–109% (lowest for 6:2 FTS). Concentrations were not corrected for recoveries. Calibration curves showed R^2^ values of 0.9868–0.9999 and generally 70–130% accuracy. Elevated calibration drift was observed for PFPS (141%), and these results were interpreted with caution. PFBA, PFHxDA, PFODA, and PFOSAA could not be quantified for analytical reasons (Table A6 and Table A8, Appendix A and Appendix B). Target-PFAS data for site G were invalid because of low internal-standard recovery.

**Figure 2:**
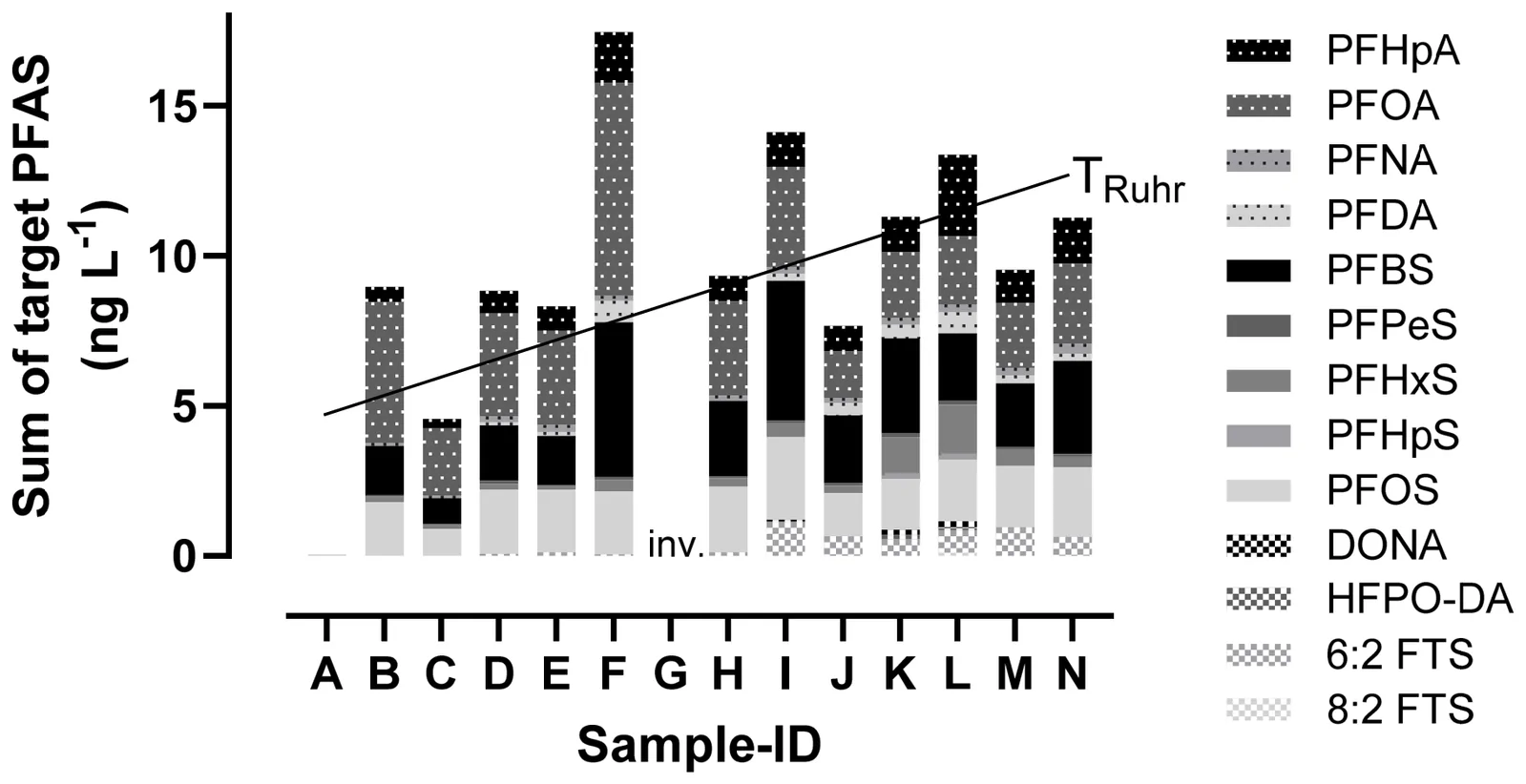
Stacked concentrations of quantified target PFASs in ng L^−1^, determined by LC-ESI-MS/MS, in surface water samples A–N. inv. = invalid data. T_Ruhr_ = simple linear regression for the sum of PFAS concentrations in samples of the river Ruhr (except F = river Möhne and G).

Thirteen PFASs were detected and quantified: four perfluoro carboxylic acids (PFHpA, PFOA, PFNA, and PFDA), five perfluoro sulfonic acids (PFBS, PFPS, PFHxS, PFHpS, and PFOS), and four other PFAS (DONA, HFPO-DA, 6:2 FTS, and 8:2 FTS) (Figure 2 and Table A8, Appendix B). No sulfonamides or sulfonamidoacetic-acid-related compounds were detected above the LOD.

The highest sum of quantified PFASs was measured in the Möhne sample (F), at 17.5 ng L^−1^, dominated by PFOA, PFBS, PFOS, and PFHpA. Along the river Ruhr, both the sum of target-PFAS concentrations and the number of detected PFAS increased downstream (simple linear regression, T_Ruhr_; Figure 2 and Table A8, Appendix B). At the source (A), only PFOS was detected, at 0.06 ng L^−1^. At most other Ruhr sites, PFHpA, PFOA, PFNA, PFBS, PFPS, PFHxS, and PFOS were detected. Concentrations >1.0 ng L^−1^ occurred for PFHpA, PFOA, PFBS, PFHxS, PFOS, and 6:2 FTS. The highest sums in Ruhr samples occurred at I (14.1 ng L^−1^), L (13.4 ng L^−1^), K (11.3 ng L^−1^), and N (11.2 ng L^−1^). DONA, HFPO-DA, and 6:2 FTS were detected more frequently downstream, and site I showed the highest 6:2 FTS concentration (1.14 ng L^−1^).

### 3.3. Extractable Organic Fluorine (EOF)

**Figure 3:**
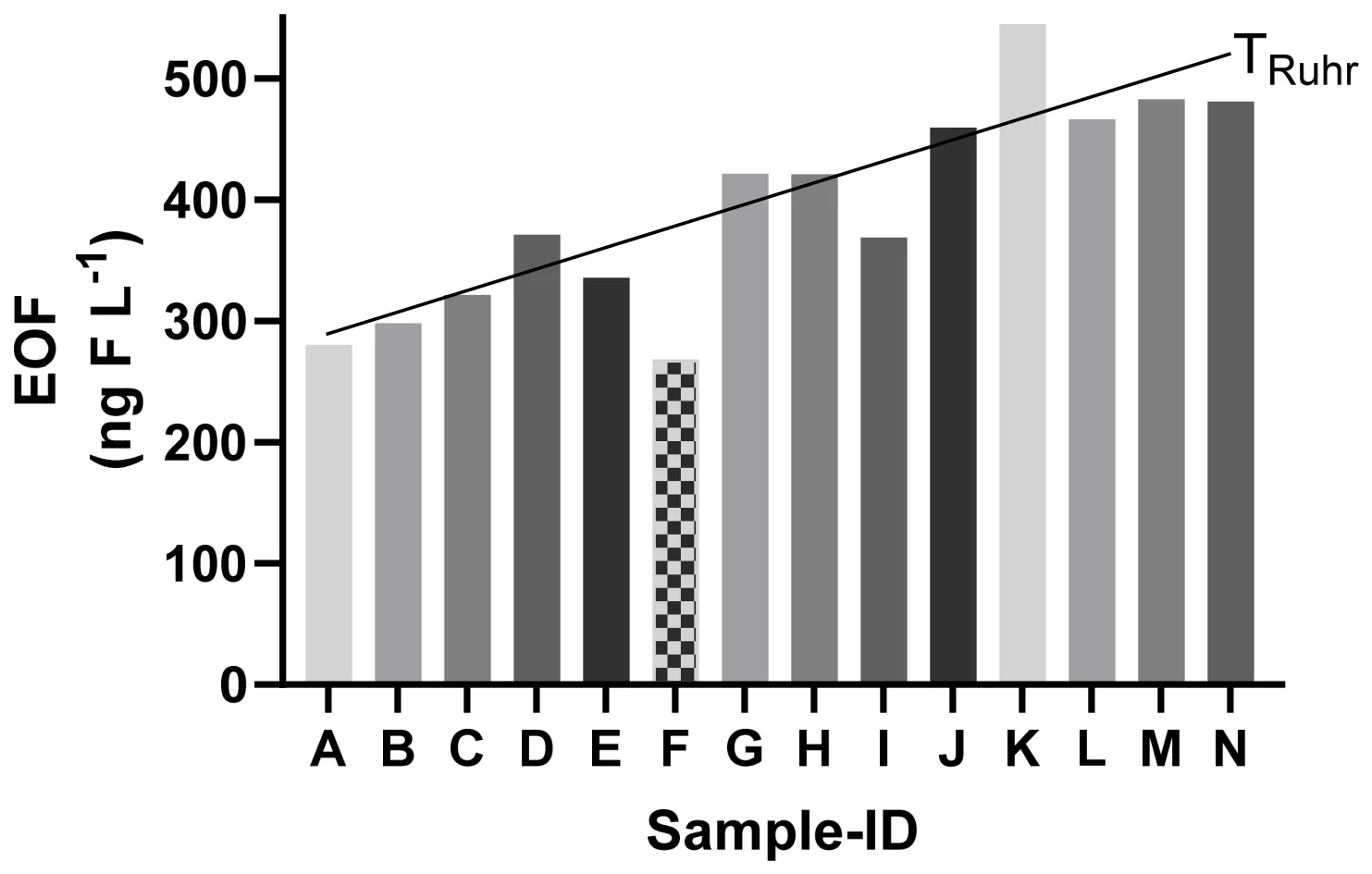
Extractable organic fluorine (EOF) in ng F L^−1^, determined by CIC, in surface water samples A–N. T_Ruhr_ = simple linear regression for EOF in samples of the river Ruhr (except F = river Möhne).

The content of extractable organic fluorine in ng fluorine (F) L^−1^ measured by CIC in all surface water samples (A–N) is shown in Figure 3. In the river Ruhr, EOF levels constantly increased downstream the flow path of the river Ruhr from 280 ng F L^−1^ at location A to 481 ng F L^−1^ at location N. This is also indicated by the trend analysis (T_Ruhr_) for the parameter EOF in the Ruhr samples (Figure 3). The highest EOF concentration of 545 ng F L^−1^ was detected at location K. In contrast, the sample collected from the river Möhne near its confluence with the river Ruhr showed the lowest EOF concentration (268 ng F L^−1^) observed across all investigated sites.

### 3.4. PFAS CALUX

**Figure 4:**
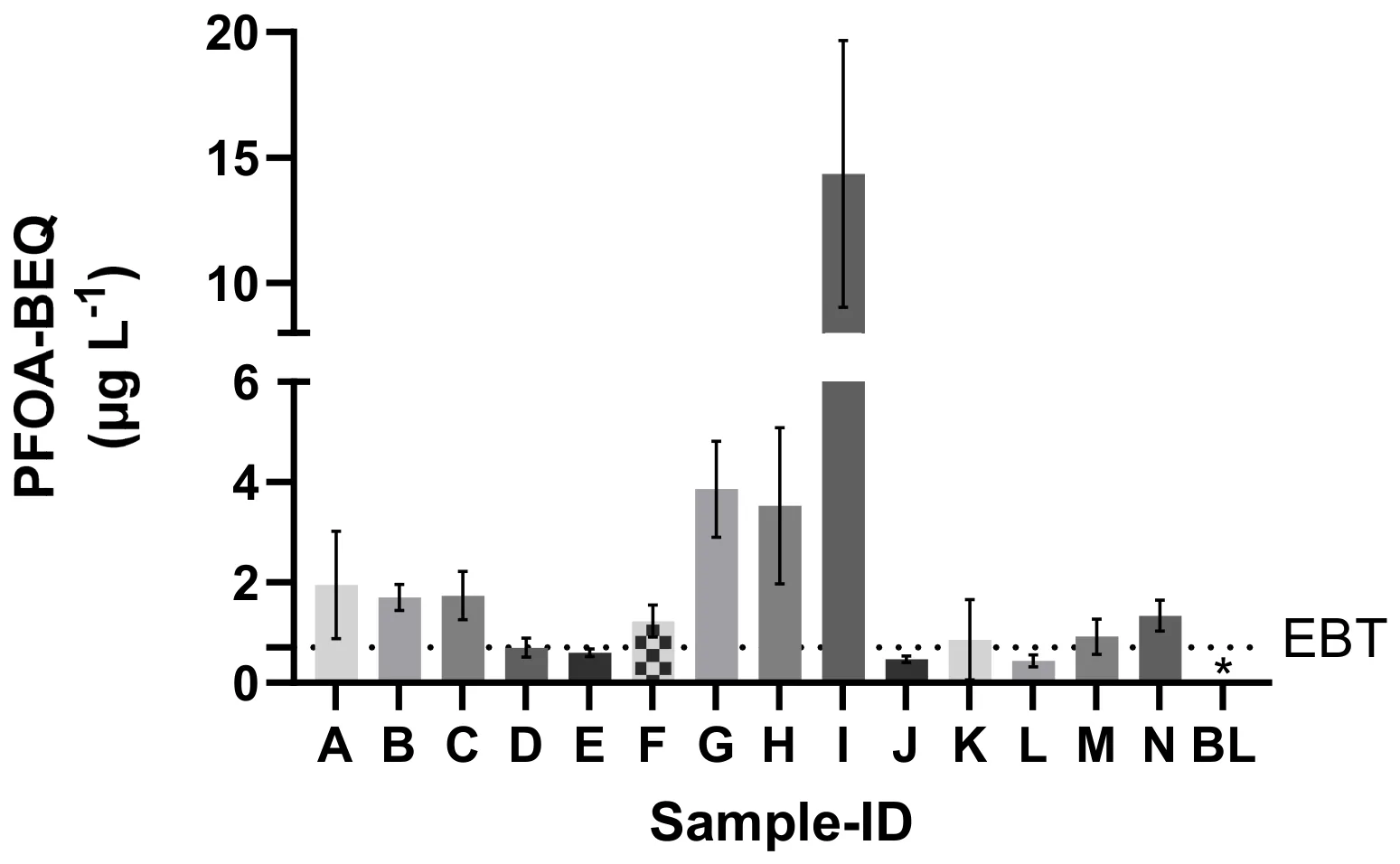
PFOA biological-equivalence concentrations (PFOA-BEQ) in µg L^−1^, determined using the PFAS CALUX bioassay, in surface water samples A–N and blank (BL). EBT = effect-based trigger value (0.71 µg PFOA-eq. L^−1^), * < LOQ.

The disruption of T4-binding to the TTR-receptor, by PFASs present in the surface water samples (A–N), is expressed as biological equivalence concentration of PFOA in µg L^−1^ (PFOA-BEQ, Figure 4). The LOQ was based on the PC_80_ of the concentration response curves of the reference compound PFOA and amounts to 0.30 µg PFOA L^−1^.

For all surface water samples PFOA-BEQ above LOQ were detected. The BEQ of the blank was below the LOQ. The BEQ from ten out of fourteen samples exceeded the EBT_PFAS CALUX_ value of 0.71 µg PFOA-eq. L^−1^. The BEQ of seven surface water samples were up to three times above the EBT value. Surface water samples of sampling location G, H, and I showed the highest exceedances of the EBT value by factors of 5, 5.4, and 20.2, respectively. Interestingly, effects detected at sites G, H, and I were not visible at downstream sampling points.

### 3.5. Complementary Comparison of Target Analysis, EOF, and Effect-Based Response

#### 3.5.1. Contribution of Target PFASs to EOF

**Figure 5:**
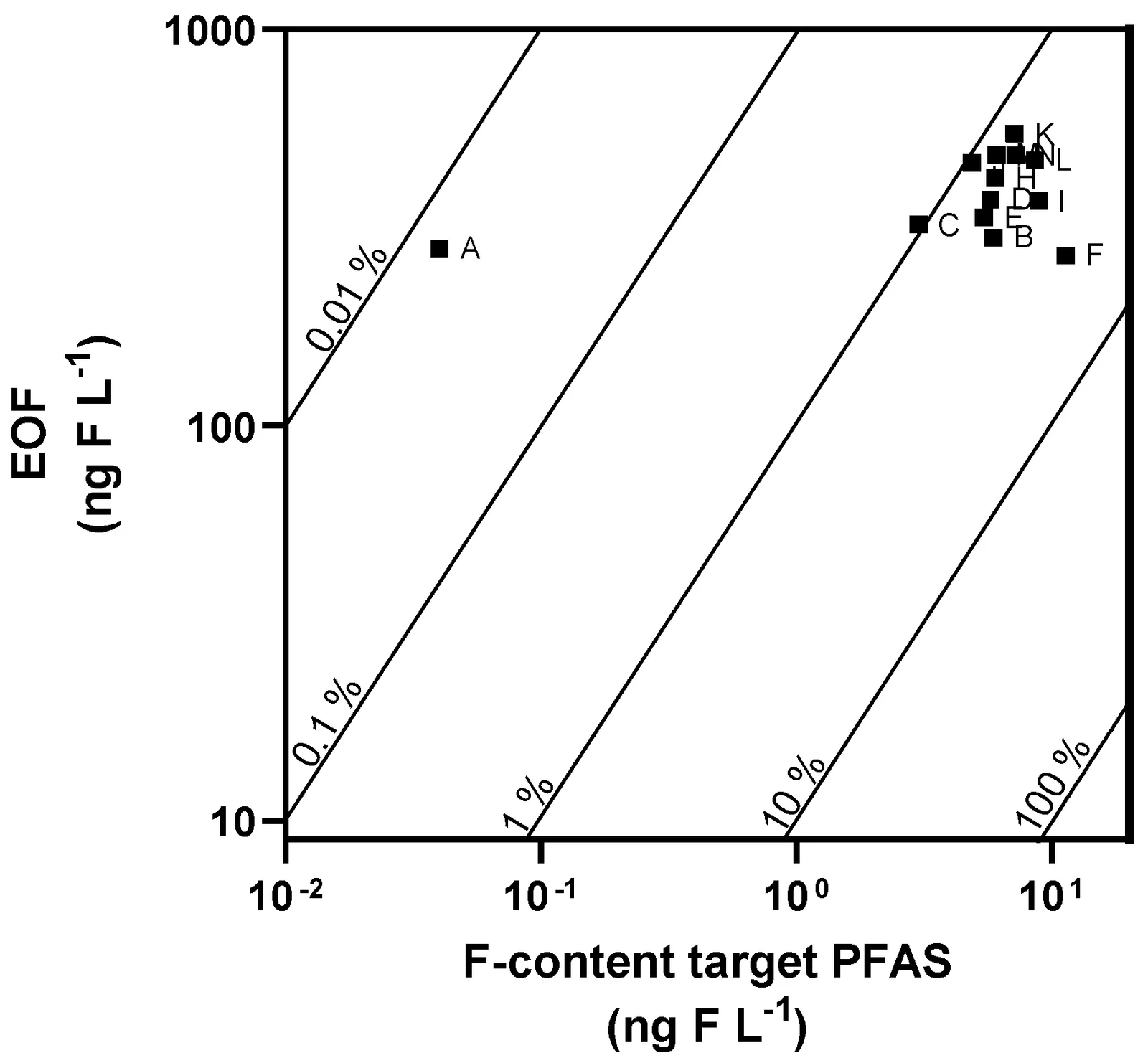
Comparison of fluorine concentrations in ng F L^−1^, detected by EOF analysis and target-PFAS analysis, for surface water samples A–N (except G = invalid data). Solid lines indicate percentages of bulk organofluorine explainable by targeted analysis.

Comparison of EOF with the fluorine content attributable to quantified target PFASs (Figure 5) showed that the target analysis explained only up to 4.2% of the measured EOF (site F, River Möhne). For river Ruhr samples, the explained fraction ranged from 0.9 to 2.4%, except at the source (A), where it was 0.014%. This large unexplained fraction is used here as contextual evidence for the environmental survey and not as a direct measure of PFAS-specific toxicity or risk.

#### 3.5.2. Contribution of Target PFASs to the PFAS CALUX Response

**Figure 6:**
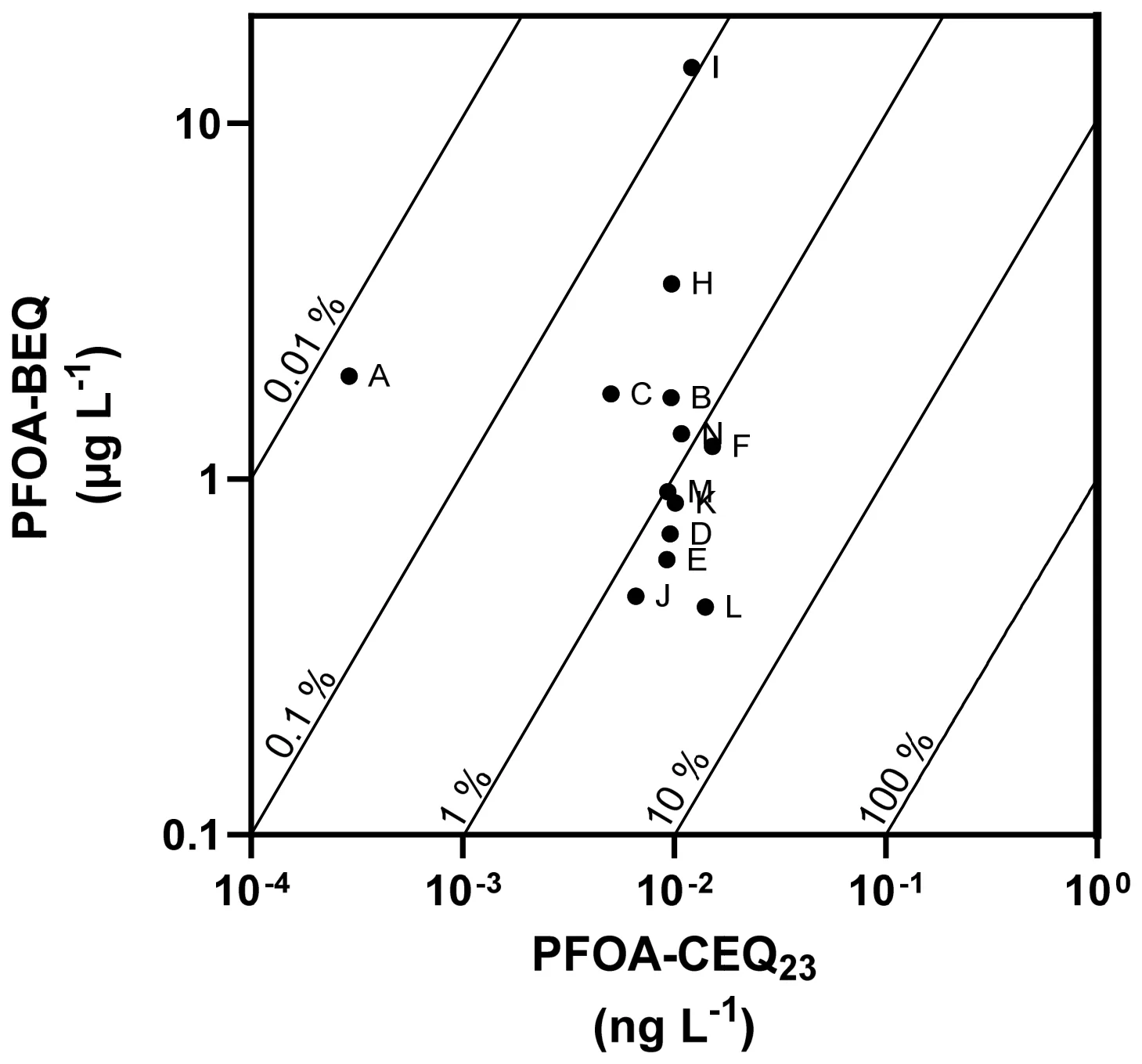
Comparison of PFOA-equivalent concentrations measured in the PFAS CALUX bioassay (PFOA-BEQ) in µg PFOA-eq. L^−1^ and by LC-ESI-MS/MS (PFOA-CEQ_23_) in ng PFOA-eq. L^−1^ for surface water samples A–N (except G = invalid data). Solid lines indicate percentages of biological effect explainable by targeted analysis.

Iceberg modelling was used to estimate how much of the PFAS CALUX response could be accounted for by target-PFAS data. PFOA-BEQ from the bioassay was compared with PFOA-CEQ_23_ derived from chemical analysis. The BEQ is expressed in µg PFOA-eq. L^−1^ and CEQ_23_ in ng PFOA-eq. L^−1^. For most sites, the target-PFAS estimate accounted for approximately 1% of the measured bioassay response; at sites I and A it accounted for <0.1% (Figure 6). The remaining response therefore cannot be assigned to the quantified target PFASs by this comparison.

### 3.6. Comparison of Chemical and Effect-Based PFAS Risk-Screening Criteria

#### 3.6.1. PFOS Concentrations Relative to the Former PFOS AA-EQS

**Figure 7:**
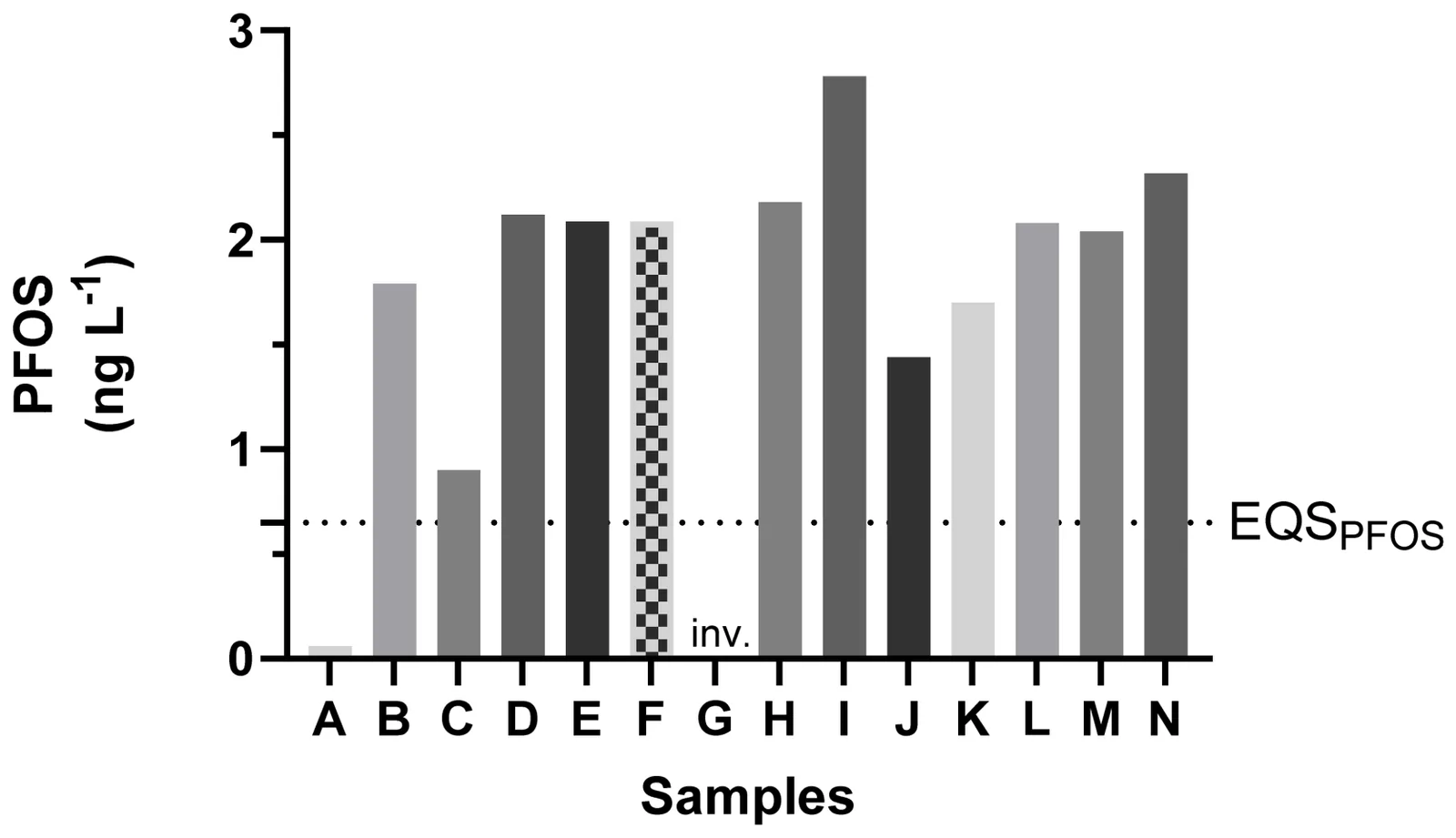
PFOS concentrations (ng L^−1^) in surface-water samples A–N compared with the former PFOS annual average EQS of 0.65 ng L^−1^. inv. = invalid target-PFAS data.

Except for site A (0.06 ng PFOS L^−1^), PFOS concentrations exceeded the former PFOS AA-EQS of 0.65 ng L^−1^ at every location with valid target-PFAS data (Figure 7). Except for sites A and C, PFOS concentrations were at least twice the criterion. The highest PFOS concentration occurred at site I (2.78 ng L^−1^), corresponding to 4.3 times the PFOS AA-EQS.

#### 3.6.2. PFOA-TEQ_WFD_ Relative to the Revised Sum-of-25-PFASs Water EQS

**Figure 8:**
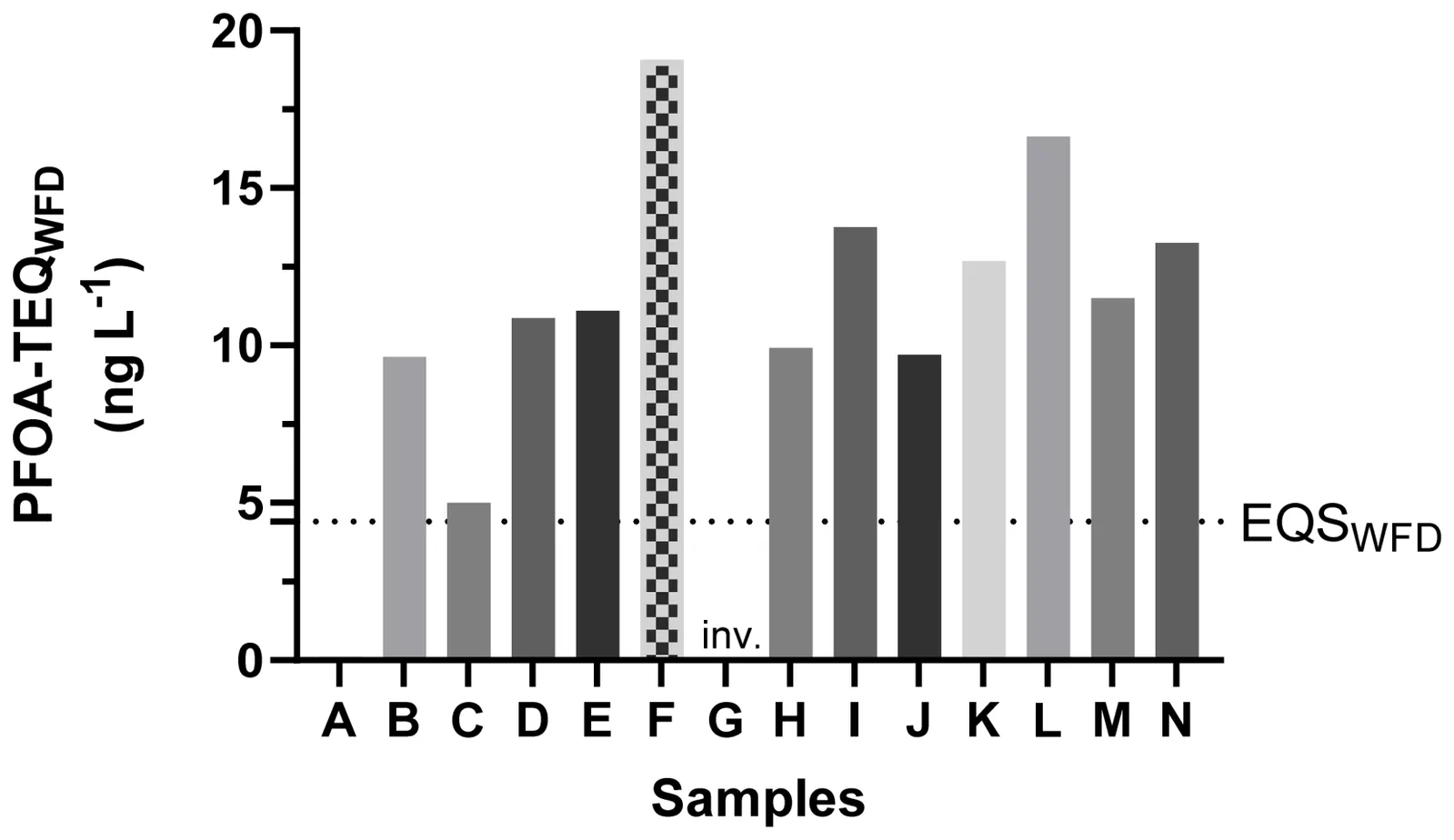
In vivo RPF-based PFOA-TEQ_WFD_ (ng PFOA-eq. L^−1^) for surface-water samples A–N compared with the revised annual average water EQS of 4.4 ng PFOA-eq. L^−1^ for the sum of 25 PFASs. PFOA-TEQ_WFD_ is a lower-bound estimate because TFA and C6O4 were not measured. inv. = invalid target-PFAS data.

The lower-bound PFOA-TEQ_WFD_ exceeded the revised water EQS of 4.4 ng PFOA-eq. L^−1^ at every location with valid target-PFAS data except site A (Figure 8). Thus, the binary exceedance classification was the same as for the former PFOS criterion. Except for sites A and C, PFOA-TEQ_WFD_ values were at least twice the revised water EQS. The highest TEQ_WFD_ was calculated for site F (River Möhne), at 19.1 ng PFOA-eq. L^−1^, corresponding to 4.3 times the EQS. Because TFA and C6O4 were not measured, the reported PFOA-TEQ_WFD_ values may underestimate the complete sum-of-25-PFASs PFOA-equivalent concentration.

#### 3.6.3. PFOA-BEQ Relative to the PFAS CALUX Effect-Based Trigger Value

The comparison of PFOA-BEQ with the pragmatic PFAS CALUX EBT of 0.71 µg PFOA-eq. L^−1^ is presented in Section 3.4. Ten of the fourteen samples exceeded this effect-based screening threshold.

#### 3.6.4. Effect of the Revised Chemical Criterion on Site Classification and Comparison with PFAS CALUX

**Figure 9:**
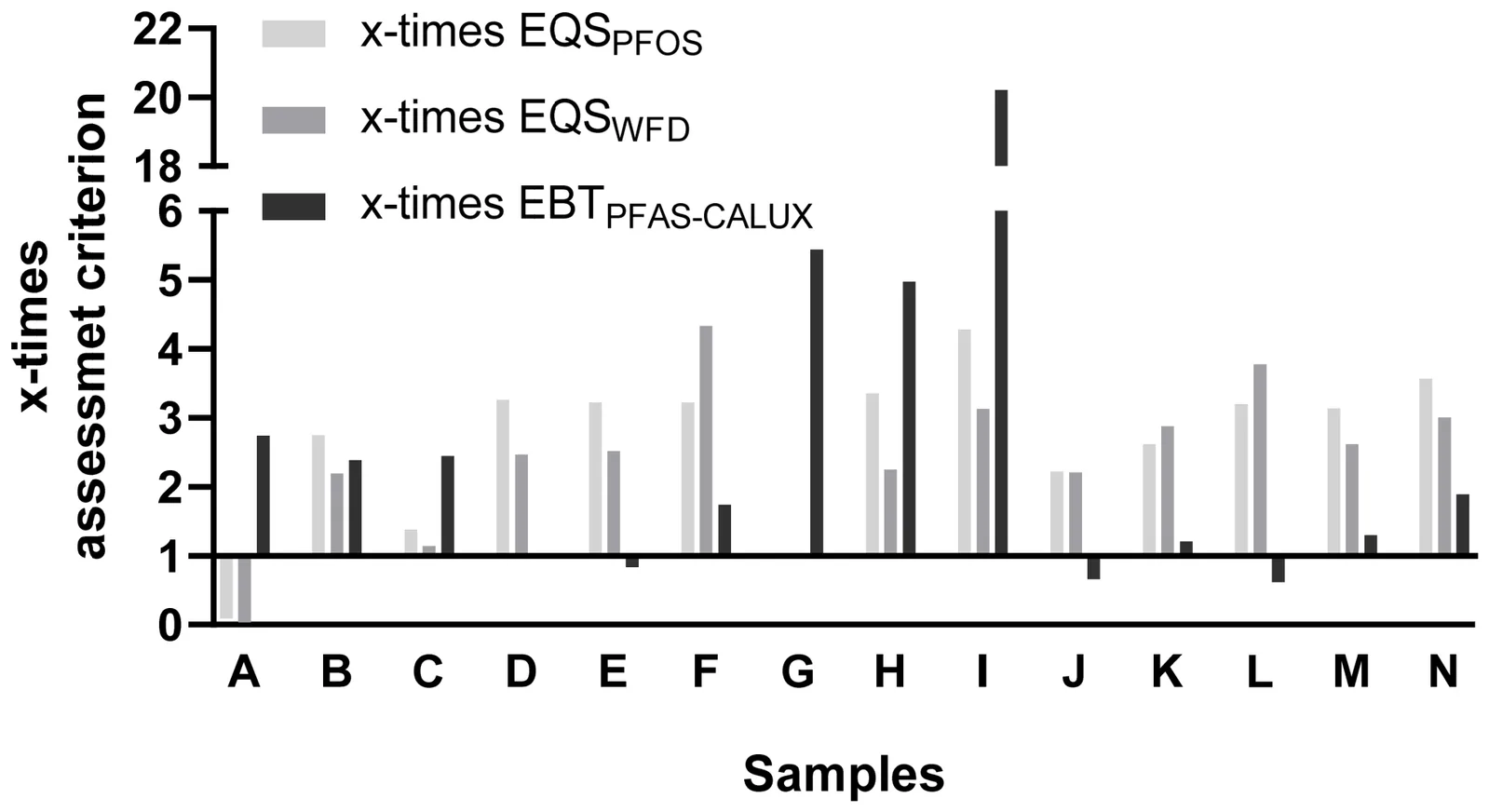
Screening-level comparison of PFAS assessment approaches in surface waters. Bars show the ratio to the former PFOS AA-EQS, the revised sum-of-25-PFASs AA-EQS (using the lower-bound PFOA-TEQ_WFD_), and the PFAS CALUX EBT. A ratio of 1 indicates the respective assessment threshold.

Figure 9 compares the ratios to the three assessment criteria (PFOS AA-EQS, the revised sum-of-25-PFASs AA-EQS, and the PFAS CALUX EBT). Transition from the former PFOS criterion to the revised potency-weighted PFAS metric did not change the binary chemical screening classification at any site for which target-PFAS data were valid: site A was below both chemical criteria, whereas the remaining valid sites exceeded both. The effect-based assessment produced a different pattern. At site A, PFAS CALUX exceeded the EBT, although both chemical criteria were met; at sites D, E, J, and L, the chemical criteria were exceeded, whereas the PFAS CALUX response remained below the EBT.

## 4. Discussion

The principal finding of this environmental survey is that the regulatory transition from a PFOS-only criterion to a potency-weighted PFAS mixture did not materially change the chemical screening classification of the sampled river Ruhr system. At every site with valid target-PFAS data except A, both the former PFOS AA-EQS and the revised sum-of-25-PFASs water criterion were exceeded. Thus, although the revised framework broadens the chemical basis of assessment, its implementation in this dataset does not by itself identify a different set of sites of concern. This is important for interpreting the practical consequence of the revised WFD: broader chemical coverage can improve attribution and future robustness without necessarily changing a binary exceedance outcome where PFOS and other potent PFASs already drive the assessment.

The occurrence data provide the environmental context for this comparison. Thirteen PFASs were detected and quantified, with PFHpA, PFOA, PFBS, PFHxS, and PFOS among the dominant compounds. Concentrations and downstream trends were broadly consistent with routine monitoring reported by the Ruhrverband [17], although direct comparison is limited by differences in sampling dates, sites, target lists, and treatment of values below detection or quantification limits. The Möhne sample showed particularly high concentrations of several PFASs, while along the Ruhr the number and summed concentration of detected PFASs generally increased downstream. These observations support the use of the dataset as a realistic survey of a river receiving numerous municipal, industrial, and rain-related discharges.

The incomplete coverage of the revised sum-of-25-PFASs metric is particularly relevant for TFA. TFA is included in the revised EU surface-water PFAS group [7] but was not determined by the C18-based target method used here; C6O4 was likewise not measured. PFOA-TEQ_WFD_ should therefore be regarded as a lower-bound regulatory estimate. An illustrative calculation based on an independently reported average TFA concentration of approximately 1.9 µg L^−1^ for the monitoring location corresponding to site L [17] and an RPF of 0.002 yields approximately 3.8 ng PFOA-eq. L^−1^ from TFA alone. This value is close to the 4.4 ng PFOA-eq. L^−1^ water EQS and demonstrates why TFA can materially affect the revised metric. Because the TFA value was not measured in the samples analyzed in this study, it should not be added quantitatively to the site-specific PFOA-TEQ_WFD_. Importantly, missing positive contributions cannot reverse the exceedances already observed at sites above the EQS, but they could affect the classification and exceedance magnitude at sites close to or below the threshold, especially site A.

Due to the constant increase of EOF in the river Ruhr PFAS hotspot detection using EOF, compared to a study in Berlin [9], was not possible. This might be explained by the huge number of direct discharges at the river Ruhr compared to more centralized discharges via WWTP in Berlin. Consequently, the use of EOF analysis for the identification of hotspots in the river Ruhr requires higher-resolution sampling to separate between several potential direct discharges. The downstream increase in EOF also translates into a substantial organofluorine mass flux. Using mean annual discharges at Meschede, Neheim, Wetter, and Mülheim together with the corresponding EOF concentrations, approximate annual EOF loads of 0.08, 0.29, 0.95, and 1.11 t year^−1^ were obtained. The estimated contribution of the Ruhr to the Rhine EOF load is on the order of 10–15% because of the much larger Rhine discharge [28,29]. In comparison, the fluorine mass represented by quantified target PFASs corresponds to only about 22 kg year^−1^ at the river mouth. These calculations reinforce the presence of a large uncharacterized fluorine mass, but they should be interpreted as contaminant-load information rather than direct evidence of ecological risk.

The effect-based results provide a different perspective from the chemical EQS-based assessment. The PFAS CALUX and chemical criteria were not always concordant, with both false-positive and false-negative classifications relative to each other across the sites investigated. PFOA-BEQ ranged from 0.4 to 14.4 µg PFOA-eq. L^−1^, and 10 of 14 samples exceeded the pragmatic PFAS CALUX EBT. The strongest responses occurred at sites G, H, and I, with site I exceeding the EBT by approximately twenty-fold. Chemically derived PFOA-CEQ_23_ accounted for only a small fraction of the bioassay response, generally around 1% and <0.1% at sites A and I, consistent with previous observations in surface waters, and has already been discussed in that context [13]. More importantly for the present regulatory comparison, the bioassay and chemical criteria were not always concordant: site A was below both chemical thresholds but above the EBT, whereas sites E, J, and L exceeded the chemical criteria but not the EBT. One reason for the differences between the assessment criteria is the number of compounds included in the assessment (PFOA or 25 PFASs vs. all possible bioactive compounds, potentially including some non-PFASs). Additionally, toxicological endpoints and therefore in vivo and in vitro RPFs largely differ for some compounds (Table A3, Appendix A). Therefore, and because the PFAS CALUX EBT is pragmatic and mechanism-specific, the effect-based assessment should not be interpreted as equivalent to a regulatory EQS; nevertheless, the discordance demonstrates that the chemical and effect-based approaches provide different information about the sampled mixtures.

The marked decrease in PFOA-BEQ downstream of sites G–I may be influenced by the Hengstey and Harkort reservoirs between sites I and J. Reservoirs can modify particle transport, sedimentation, and biogeochemical processing [30,31], and longer-chain PFASs generally have greater particle affinity than short- and ultra-short-chain compounds [32]. Preferential removal of more strongly TTR-active long-chain PFASs could therefore contribute to the observed decrease in bioassay response. The concurrent decrease in the sum of detected PFASs downstream of the reservoirs is consistent with this interpretation. EOF did not show the same decrease. Most probably due to degradation to short-chain PFASs in the reservoirs. This mechanistic explanation remains a hypothesis because (ultra)-short-chain PFASs, sediment, suspended-particle, and transformation data were not collected in the present survey.

Taken together, the three approaches provide complementary information. The revised sum-of-25-PFASs EQS broadens the chemically defined regulatory assessment, while PFAS CALUX identifies biological activity that is not necessarily reflected by the target-PFAS concentrations, and EOF indicates the magnitude and spatial distribution of the wider extractable organofluorine burden. Complete application of the revised regulatory metric will require inclusion of TFA and C6O4. Repeated seasonal sampling and complementary analysis of sediment and biota would help determine the persistence and environmental relevance of the spatial patterns observed in this survey.

## 5. Conclusions

Applying the former PFOS AA-EQS and the revised 4.4 ng PFOA-eq. L^−1^ sum-of-25-PFASs water criterion to the river Ruhr dataset resulted in the same binary chemical screening classification. All locations with valid target-PFAS data except site A exceeded both criteria. Thus, for the sites investigated here, expanding the regulatory assessment from PFOS alone to a potency-weighted PFAS mixture did not identify additional sites of concern. Because TFA and C6O4 were not included in the analytical method, the calculated PFOA-TEQ values represent lower-bound estimates of the complete revised metric.

The effect-based assessment provided a different picture. Ten of 14 samples exceeded the pragmatic PFAS CALUX effect-based trigger value, and several sites showed discordant chemical and effect-based classifications. In addition, quantified target PFASs accounted for less than 5% of the measured EOF and generally only a small fraction of the PFAS CALUX response. These results show that neither the regulatory target-PFASs assessment nor EOF or PFAS CALUX alone fully characterizes the chemical and biological properties of the sampled mixtures.

For the river Ruhr, the results therefore demonstrate that the revised PFASs EQS provides a broader chemical assessment without substantially changing the site classification observed with PFOS alone, whereas EOF and PFAS CALUX provide complementary information on uncharacterized organofluorine and biological activity. Sites at which chemical and effect-based assessments diverge represent particularly relevant candidates for further chemical characterization and follow-up monitoring.

## Data Availability

The raw data supporting the conclusions of this article will be made available by the authors on request.

## Abbreviations

The following abbreviations are used in this manuscript:

PFASs	per- and polyfluoroalkyl substance
PFAS acronyms	See Table A3
EU	European Union
WFD	Water Framework Directive
PFOS	perfluorooctane sulfonic acid
AA-EQS	annual average environmental quality standard
EQS	environmental quality standard
PFOA	perfluorooctanoic acid
RPF	relative potency factors
PFOA-eq.	perfluorooctanoic acid equivalents
EOF	extractable organic fluorine
TTR	transthyretin (thyroxine transport protein)
T4	thyroxine (thyroid hormone)
BEQ	biological equivalence concentrations
LC	liquid chromatography
ESI	electrospray ionization
MS	mass spectrometry
CIC	combustion ion chromatography
EBT	effect-based trigger value
WWTP	wastewater treatment plant
MQ	mean annual discharge
W	water level
Q	discharge
WAX	weak anion exchange
SPE	solid phase extraction
R^2^	coefficient of determination
LOD	limit of detection
LOQ	limit of quantification
TRβ	thyroid-receptor
CALUX	chemical activated luciferase gene expression
PC80	concentration at 80% sample activity compared to PFOA response
TFA	trifluoroacetic acid
C6O4	2,2-difluoro-2-([2,2,4,5-tetrafluoro-5-(trifluoromethoxy)-1,3-dioxolan-4-yl]oxy)acetic acid
PFOA-EQ	PFOA-equivalent concentrations
PFOA-BEQ	PFOA biological equivalence concentrations (determined with bioassay)
PFOA-TEQ	PFOA-equivalent concentrations (calculated using in vivo RPF)
PFOA-CEQ	PFOA-equivalent concentrations (calculated using in vitro RPF)
T_Ruhr_	trend of parameter in the river Ruhr (simple linear regression)
F	fluorine
SD	standard deviation

## Appendix A

Appendix A contains supplementary information on sampling locations (Table A1), on the sample preparation technique (Table A2), and on the investigated PFAS (Table A3), as well as on the LC MS method for PFAS quantification (Table A4 and Table A5).

**Table A1:** Sample-ID, sampling location, coordinates (latitude, longitude), surface water, date of sampling, and corresponding location of the regular Ruhr monitoring on organic trace substances.

Sample-ID	Sampling Location	Coordinates Lat., Long.	Surface Water	Date of Sampling	River km	Corresponding Location Regular Monitoring
A	Ruhrquelle	51.213721, 8.558250	Ruhr	22 May 2024	219.14	Unterhalb Quelle
B	Fahrradbrücke Bestwig	51.358157, 8.382618	Ruhr	22 May 2024	190.29	Unterhalb Valme
C	Ruhrbrücke Wennemen	51.346330, 8.205176	Ruhr	22 May 2024	176.12	Unterhalb Meschede
D	Brücke Oeventrop	51.390906, 8.130562	Ruhr	22 May 2024	160.72	Brücke Oeventrop
E	R-Cafe Neheim	51.440605, 7.963181	Ruhr	22 May 2024	139.59	Ruhr oberhalb des Möhnezuflusses
F	Möhnebrücke Neheim	51.455815, 7.957776	Möhne	22 May 2024	0.27	Möhne im Mündungsbereich
G	Ruhrbrücke Echthausen	51.487815, 7.924566	Ruhr	22 May 2024	131.81	Brücke Echthausen
H	Ruhrbrücke Langschede	51.473039, 7.709776	Ruhr	22 May 2024	111.93	Ü-Station Fröndenberg
I	Ruhrbrücke Westhofen	51.414394, 7.505726	Ruhr	29 May 2024	93.74	Kraftwerk Westhofen
J	Pegel Wetter	51.383335, 7.383746	Ruhr	29 May 2024	80.66	Pegel Wetter
K	DLRG Hattingen	51.388310, 7.162048	Ruhr	29 May 2024	55.63	Ü-Station Hattingen
L	Konrad-Adenauer Brücke Essen-Rellinghausen	51.427486, 7.056324	Ruhr	29 May 2024	42.44	Zornige Ameise
M	Ruhrbrücke Essen Werden	51.387155, 6.999739	Ruhr	29 May 2024	29.91	Ü-Station Essen
N	Sliprampe Dusiburg Hafeneinfahrt	51.447561, 6.724341	Ruhr	29 May 2024	0.38	Ü-Station Duisburg

**Table A2:** Comparison of major steps in weak anion exchange solid phase extraction (WAX-SPE) as sample preparation for the analysis of surface water samples by EOF, target PFASs, and PFAS CALUX.

WAX-SPE Steps	EOF	Target PFAS	PFAS CALUX
Conditioning	4 mL 0.1% ammonium hydroxide in methanol; 4 mL methanol; 4 mL ultrapure water 4 mL of methanol, 4 mL ultrapure water		
Sample volume loaded	500 mL	1000 mL spiked with 10 µL of 16–40 µg L^−1^ MPFAC-HIF-ES mix	1000 mL
Washing	4 mL 0.1% ammonium acetate in ultra pure water		
Elution	4 mL methanol; 4 mL 0.1% ammonium hydroxide in methanol		
Reconstitution	500 µL of methanol, 500 µL of ultrapure water	1000 µL methanol	50 µL dimethyl sulfoxide
Concentration factor	500	1000	20,000

**Table A3:** List of analyzed PFASs with acronyms sorted by substance groups. Where available, in vitro derived relative potency factors (RPFs) and in vivo derived RPFs are given, as well as method LOD/LOQ in ng L^−1^ and recovery rate in %. na = not analyzable.

PFAS Group/PFAS Name	Acronym	In Vitro RPF (Sosnowska et al. 2025 [15])	In Vivo RPF (Directive (EU) 2026/805)	Method LOD (ng L^−1^)	Method LOQ (s/n > 10) (ng L ^−1^)	Recovery (%)
PFAS carboxylic acids						
Perfluorobutanoic acid	PFBA	0.0019	0.05	na	na	na
Perfluoropentanoic acid	PFPeA	0.083	0.03	0.011	0.037	82
Perfluorohexanoic acid	PFHxA	0.19	0.01	0.003	0.011	53
Perfluoroheptanoic acid	PFHpA	1.5	0.505	0.003	0.011	87
Perfluorooctanoic acid	PFOA	1	1	0.011	0.037	74
Perfluorononanoic acid	PFNA	0.34	10	0.011	0.037	86
Perfluorodecanoic acid	PFDA	0.13	7	0.011	0.037	109
Perfluoroundecanoic acid	PFUnDA	0.001	4	0.011	0.037	77
Perfluorododecanoic acid	PFDoDA	0.025	3	0.035	0.116	83
Perfluorotridecanoic acid	PFTrDA	-	1.65	0.011	0.037	88
Perfluorotetradecanoic acid	PFTeDA	-	0.3	0.003	0.011	81
Perfluorohexadecanoic acid	PFHxDA	-	0.02	na	na	na
Perfluorooctadecanoic acid	PFODA	-	0.02	na	na	na
PFAS sulfonic acids						
Perfluoro-1-butanesulfonic acid	PFBS	0.054	0.001	0.011	0.037	90
Perfluoro-1-pentanesulfonic acid	PFPS	0.082	0.3005	0.011	0.037	87
Perfluoro-1-hexanesulfonic acid	PFHxS	1.6	0.6	0.011	0.037	73
Perfluoro-1-heptanesulfonic acid	PFHpS	1	1.3	0.011	0.037	86
Perfluoro-1-octanesulfonic acid	PFOS	2.1	2	0.011	0.037	80
Perfluoro-1-nonanesulfonic acid	PFNS	0.0045	-	0.011	0.037	101
Perfluoro-1-decanesulfonic acid	PFDS	<0.0001	2	0.011	0.037	84
PFAS sulfonamides and sulfonamidoacetic acids						
Perfluoro-1-octanesulfonamide	PFOSA	0.75	-	0.011	0.037	42
Perfluorooctanesulfonamidoacetate	PFOSAA	<0.0001	-	na	na	na
N-ethyl perfluorooctyl sulfonamide	EtFOSA	0.054	-	0.003	0.011	90
2-(N-Methylperfluoro-1-octanesulfonamido)-ethanol	MeFOSE	-	-	0.003	0.011	90
2-(N-ethylperfluoro-1-octanesulfonamido)-ethanol	EtFOSE	-	-	0.011	0.037	97
Other PFAS						
2,2,3-trifluoro-3-(1,1,2,2,3,3-hexafluoro-3-(trifluoromethoxy)propoxy)propanoic acid	DONA	0.55	0.03	0.011	0.037	104
2,3,3,3-tetrafluoro-2-(heptafluoropropoxy)propionic acid	HFPO-DA	0.0039	0.06	0.003	0.011	104
4:2 Fluorotelomer sulfonic acid	4:2 FTS	0.018	-	0.003	0.011	118
6:2 Fluorotelomer sulfonic acid	6:2 FTS	0.02	0.02	0.011	0.037	67
8:2 Fluorotelomer sulfonic acid	8:2 FTS	-	0.04	0.011	0.037	90

**Table A4:** List of monitored transitions and MS parameters.

Compound Name	Precursor Ion (m/z)	Product Ion (m/z)	Ret Time (min)	Fragmentor	Collision Energy (V)	Polarity
4_2 FTS	327	307	6.3	94	20	Negative
4_2 FTS	327	81	6.3	94	36	Negative
6_2 FTS	427	407	7.7	128	24	Negative
6_2 FTS	427	81	7.7	128	56	Negative
8_2FTS	527	507	9.7	134	28	Negative
8_2FTS	527	81	9.7	134	68	Negative
9 Cl-PF3ONS	531	351	11.4	132	28	Negative
9 Cl-PF3ONS	531	83	11.4	132	32	Negative
10_2FTS	627	607	13.7	168	36	Negative
10_2FTS	627	81	13.7	168	68	Negative
11 Cl-PF3OUdS	631	451	14	162	32	Negative
11 Cl-PF3OUdS	631	83	14	162	36	Negative
d EtFOSA	531	169	13.2	132	32	Negative
d MeFOSA	515	169	12	134	32	Negative
d3 MeFOSE	573	419	10.7	94	20	Negative
d5 EtFOSAA	589	419	11.4	94	20	Negative
d7 MeFOSE	623	59	19.9	96	52	Negative
d9 EtFOSE	639	59	20.3	96	68	Negative
FBSA	298	78	7.7	94	32	Negative
FHxSA	398	78	14.1	94	60	Negative
FOSAA	556	498	11.5	136	32	Negative
FOSAA	556	419	11.5	136	28	Negative
HFPO-DA	285	185	6.6	64	16	Negative
HFPO-DA	285	169	6.6	64	4	Negative
M2 4_2 FTS	329	309	6.3	94	20	Negative
M2 6_2 FTS	429	409	7.7	130	24	Negative
M2 8_2 FTS	529	509	9.7	162	28	Negative
M2PFOA	415	370	7.9	70	4	Negative
M2PFTeDA	715	670	15.5	100	8	Negative
M3HFPO-DA	287	169	6.6	50	4	Negative
M3PFBA	216	172	3.7	50	4	Negative
M3PFBS	302	80	6.5	130	48	Negative
M3PFHxS	402	80	8.2	132	68	Negative
M4PFHpA	367	322	7.1	66	4	Negative
M5PFHxA	318	273	6.5	64	4	Negative
M5PFPeA	268	223	6	50	4	Negative
M6PFDA	519	474	11	130	16	Negative
M8PFOA	421	376	7.9	76	8	Negative
M8PFOS	507	80	10.2	164	92	Negative
M9PFNA	472	427	9	70	8	Negative
MPFBA	217	172	3.7	50	4	Negative
MPFDA	515	470	11	168	104	Negative
MPFdDA	615	570	12.8	96	8	Negative
MPFOS	503	80	11.8	162	92	Negative
DONA	377	251	7.4	68	8	Negative
DONA	377	85	7.4	68	44	Negative
EtFOSA	526	219	20.6	132	28	Negative
EtFOSA	526	169	20.6	132	32	Negative
EtFOSE	584	483	11.4	94	12	Negative
EtFOSE	584	419	11.4	94	20	Negative
EtFOSE-M	630	59	20.5	98	68	Negative
MeFOSE	570	483	10.7	94	16	Negative
MeFOSE	570	419	10.7	94	20	Negative
MeFOSE-M	616	59	20.1	98	76	Negative
PFBA	213	169	3.7	40	4	Negative
PFBS	299	99	6.5	130	36	Negative
PFBS	299	80	6.5	130	52	Negative
PFDA	513	469	11	74	8	Negative
PFDA	513	219	11	74	16	Negative
PFDoDA	613	529	12.8	96	8	Negative
PFdDS	699	99	16	166	116	Negative
PFdDS	699	80	16	166	116	Negative
PFDS	599	99	13.1	178	88	Negative
PFDS	599	80	13.1	178	108	Negative
PFHpA	363	319	7.1	66	4	Negative
PFHpA	363	169	7.1	66	16	Negative
PFHpS	449	99	9.3	164	60	Negative
PFHpS	449	80	9.3	164	72	Negative
PFHxA	313	269	6.5	64	4	Negative
PFHxA	313	119	6.5	64	4	Negative
PFHxDA	813	769	16.5	130	20	Negative
PFHxDA	813	169	16.5	130	32	Negative
PFHxS	399	99	8.2	136	52	Negative
PFHxS	399	80	8.2	136	72	Negative
PFNA	463	419	9	72	8	Negative
PFNA	463	169	9	72	20	Negative
PFNS	549	99	11.7	174	56	Negative
PFNS	549	80	11.7	174	108	Negative
PFOA	413	369	7.9	68	4	Negative
PFOA	413	169	7.9	68	16	Negative
PFODA	913	869	17.5	130	104	Negative
PFODA	913	169	17.5	130	104	Negative
PFOS	499	99	10.5	162	68	Negative
PFOS	499	80	10.5	162	84	Negative
PFOSA	498	78	18	130	72	Negative
PFPeA	263	219	6	50	50	Negative
PFPeA	263	169	6	50	50	Negative
PFPeS	349	99	7.2	132	40	Negative
PFPeS	349	80	7.2	132	60	Negative
PFTeDA	713	669	15.4	100	12	Negative
PFTeDA	713	169	15.4	100	32	Negative
PFTrDA	663	619	14	98	8	Negative
PFTrDA	663	169	14	98	32	Negative
PFUnDA	563	519	11.5	78	8	Negative
PFUnDA	563	119	11.5	78	48	Negative

**Table A5:** Details on LC method.

Time (min)	Phase A (%)	Phase B (%)
0	97	3
1	97	3
2	70	30
14	40	60
17.5	0	100
23.5	0	100
23.6	97	3
28	97	3

**Table A6:** Coefficients of determination (R^2^), back-calculated percentage accuracies for calibration points ranging from 0.003–44.4 ng g^−1^, and calibration-drift results (mean and standard deviation (SD)) for calibration points of 0.348 and 0.707 ng g^−1^.

PFAS		PFBS	HFPO-DA	DONA	PFHpA	PFPS	6:2 FTS	PFHxS	PFOA	PFNA	PFHpS	8:2 FTS	PFOS	PFDA
Coefficient of determination	R^2^	0.9993	0.9990	0.9981	0.9868	0.9984	0.9998	0.9997	0.9999	0.9962	0.9962	0.9975	0.9998	0.9997
Back calculated accuracy (%) at calibration points (ng g^−1^)	0.003	85	75	75	106	79	92	-	-	87	87	94	118	118
	0.029	90	128	122	94	110	108	85	81	100	82	107	79	109
	0.061	108	101	101	73	100	100	113	96	117	115	97	104	104
	0.348	113	95	110	-	115	101	100	107	104	116	100	107	106
	0.707	106	102	92	81	97	99	102	96	92	99	106	103	101
	4.478	99	113	100	100	99	91	101	100	101	104	107	100	101
	8.577	99	108	98	94	93	77	99	97	82	97	98	93	105
	44.396	86	97	83	84	75	-	80	87	91	88	-	85	99
Drift (%) at calibration point 0.348 ng g^−1^	Mean	102	100	105	95	141	96	111	108	105	109	83	99	128
	SD	7.6	6.3	3.7	5.6	8.0	6.1	3.1	3.5	4.1	6.9	6.0	4.7	12
Drift (%) at calibration point 0.707 ng g^−1^	Mean	101	97	114	101	141	110	97	108	100	111	105	92	116
	SD	4.7	2.4	1.3	2.5	4.1	2.7	2.6	4.2	3.8	8.8	7.7	2.6	11

## Appendix B

Appendix B contains supplementary monitoring data such as on-site parameters (Table A7) and quantified concentrations of single PFASs, including LOD, LOQ, and recovery rates of the analytical method (Table A8).

**Table A7:** On-site parameters (temperature, conductivity, and pH) for all surface water samples (A–N).

Sample-ID	Temperature	Conductivity	pH
	°C	µS cm^−1^	
A	8.7	82	7.3
B	13.2	227	7.6
C	11.6	273	7.8
D	12.9	247	7.3
E	13.6	249	7.8
F	12.7	314	8.0
G	13.6	273	7.5
H	14.3	324	7.8
I	15.4	364	7.6
J	15.4	308	7.1
K	16.0	339	7.6
L	16.3	337	7.7
M	16.7	350	7.6
N	18.0	382	7.6

**Table A8:** Concentration of quantified target PFASs in surface-water samples (A–N) in ng L^−1^ (white = <LOD, light grey: <0.5 ng L^−1^, grey: >0.5–<1.0 ng L^−1^, dark grey: >1.0 ng L^−1^), including method LOD and LOQ in ng L^−1^ as well as the recovery rates (%) of the respective internal standard. inv. = data invalid. Results were not corrected with recoveries.

Sample ID	Perfluoro Carboxylic Acids				Perfluoro Sulfonic Acids					Other PFASs				Sum of Target PFAS
	PFHpA	PFOA	PFNA	PFDA	PFBS	PFPeS	PFHxS	PFHpS	PFOS	DONA	HFPO-DA	6:2 FTS	8:2 FTS	
	ng L^−1^	ng L^−1^	ng L^−1^	ng L^−1^	ng L^−1^	ng L^−1^	ng L^−1^	ng L^−1^	ng L^−1^	ng L^−1^	ng L^−1^	ng L^−1^	ng L^−1^	ng L^−1^
A	<LOD	<LOD	<LOD	<LOD	<LOD	<LOD	<LOD	<LOD	0.06	<LOD	<LOD	<LOD	<LOD	0.06
B	0.51	4.68	0.10	<LOD	1.66	0.05	0.18	<LOD	1.79	<LOD	<LOD	<LOD	<LOD	8.99
C	0.32	2.26	0.07	<LOD	0.88	<LOD	0.15	<LOD	0.90	<LOD	<LOD	<LOD	<LOD	4.57
D	0.75	3.44	0.19	0.11	1.85	0.11	0.19	<LOD	2.12	<LOD	<LOD	0.08	<LOD	8.84
E	0.83	3.13	0.23	0.14	1.65	<LOD	0.14	<LOD	2.09	<LOD	<LOD	0.12	<LOD	8.34
F	1.69	7.10	0.17	0.71	5.15	0.10	0.39	<LOD	2.09	<LOD	<LOD	0.06	<LOD	17.46
G	inv.	inv.	inv.	inv.	inv.	inv.	inv.	inv.	inv.	inv.	inv.	inv.	inv.	inv.
H	0.85	3.13	0.18	<LOD	2.53	0.06	0.29	<LOD	2.18	<LOD	<LOD	0.12	<LOD	9.33
I	1.15	3.34	0.24	0.22	4.65	0.09	0.46	<LOD	2.78	0.06	<LOD	1.14	<LOD	14.14
J	0.85	1.54	0.18	0.41	2.26	0.08	0.26	<LOD	1.44	<LOD	<LOD	0.65	<LOD	7.65
K	1.18	2.18	0.24	0.45	3.16	0.13	1.28	0.10	1.70	0.18	0.14	0.56	<LOD	11.30
L	2.72	2.27	0.27	0.70	2.24	0.14	1.70	0.11	2.08	0.22	0.08	0.75	0.10	13.40
M	1.09	2.18	0.25	0.26	2.12	0.09	0.55	<LOD	2.04	<LOD	<LOD	0.96	<LOD	9.54
N	1.54	2.67	0.33	0.23	3.11	0.09	0.35	<LOD	2.32	<LOD	<LOD	0.64	<LOD	11.20
Method LOD	0.003	0.011	0.011	0.011	0.011	0.011	0.003	0.003	0.011	0.011	0.003	0.011	0.011	
Method LOQ	0.011	0.037	0.037	0.037	0.037	0.037	0.011	0.011	0.037	0.037	0.011	0.037	0.037	
Recovery (%)	87	74	86	109	90	87	73	86	80	104	104	67	90	88
